# Elusive Unilateral Pleural Effusion: Keys to Clinching the Diagnosis

**DOI:** 10.7759/cureus.69517

**Published:** 2024-09-16

**Authors:** Yasoda Rijal, Prakash Banjade, Seema Oli, Carl Boethel, Munish Sharma

**Affiliations:** 1 Internal Medicine, Tribhuvan University Institute of Medicine, Kathmandu, NPL; 2 Medicine, Manipal College of Medical Sciences, Pokhara, NPL; 3 Internal Medicine, University of Pittsburgh Medical Center (UPMC), Harrisburg, USA; 4 Pulmonology and Critical Care, Baylor Scott and White Medical Center, Temple, USA

**Keywords:** exudative, pleural effusion, pseudoexudates, thoracentesis, transudative

## Abstract

Unilateral pleural effusions may sometimes be difficult to diagnose. The cause may vary widely, including congestive heart failure, chronic liver and kidney disease, various drugs, and underlying undiagnosed disorders of the lung and pleura. With advancements in chest imaging, new biomarkers, and less invasive methods for obtaining tissue samples, it may be possible to identify the cause of the unilateral pleural effusions whose etiology is unclear. Even reviewing patient history, re-examining pleural fluid, classifying effusions based on Light’s criteria, and ruling out pseudoexudates can help understand the cause. We aim to discuss a case of unilateral pleural effusion and, on its backdrop, discuss an approach to elusive unilateral pleural effusion.

## Introduction

Recurrent pleural effusions can present a significant challenge for patients, causing respiratory symptoms and impairing quality of life. Although serial thoracentesis can be considered, repeated need for procedures can lead to enhanced healthcare utilization and cost. As a result, definitive management strategies have been explored over the years to address these issues. Pleural infection, heart failure, and malignancy are the most common causes of pleural effusion [1]. Less common causes include chronic liver disease, kidney disease, asbestos exposure, rheumatoid arthritis, lupus pleuritis, pancreatitis, pulmonary embolism, and cardiac surgery. Rare causes involve drugs, hypothyroidism, chylothorax, and cholesterol pleural effusion. Sometimes, the cause may not be immediately apparent [1]. The diagnosis of pleural effusion requires a comprehensive approach involving clinical evaluation, imaging studies, and diagnostic procedures such as thoracentesis [2]. We have a case involving a patient with recurring pleural effusion whose cause could not be exactly determined initially despite extensive workup. A systematic approach to rule out helped us identify the underlying cause. We intend to discuss possible keys to clinching diagnosis in such cases of unilateral pleural effusion, which doesn’t seem to have an obvious etiology during the initial workup.

## Case presentation

A 77-year-old male presented with gradually progressive shortness of breath and a non-productive cough for around three weeks. He did not have fever, chest pain, orthopnea, or paroxysmal nocturnal dyspnea. His medical history included chronic obstructive pulmonary disease, congestive heart failure with preserved ejection fraction on diuretics, non-small cell lung cancer of the left lower lobe, and recurrent left-sided pleural effusion. Lung cancer was diagnosed during routine low-dose CT scan screening. It was treated with chemoradiation and was in complete remission for eight years. One year prior to our evaluation, the patient experienced hypoxic respiratory failure, and a left pleural effusion was identified first at that time at an outside facility. He had recurrent pleural effusions since then and was referred to our institution.

**Figure 1:**
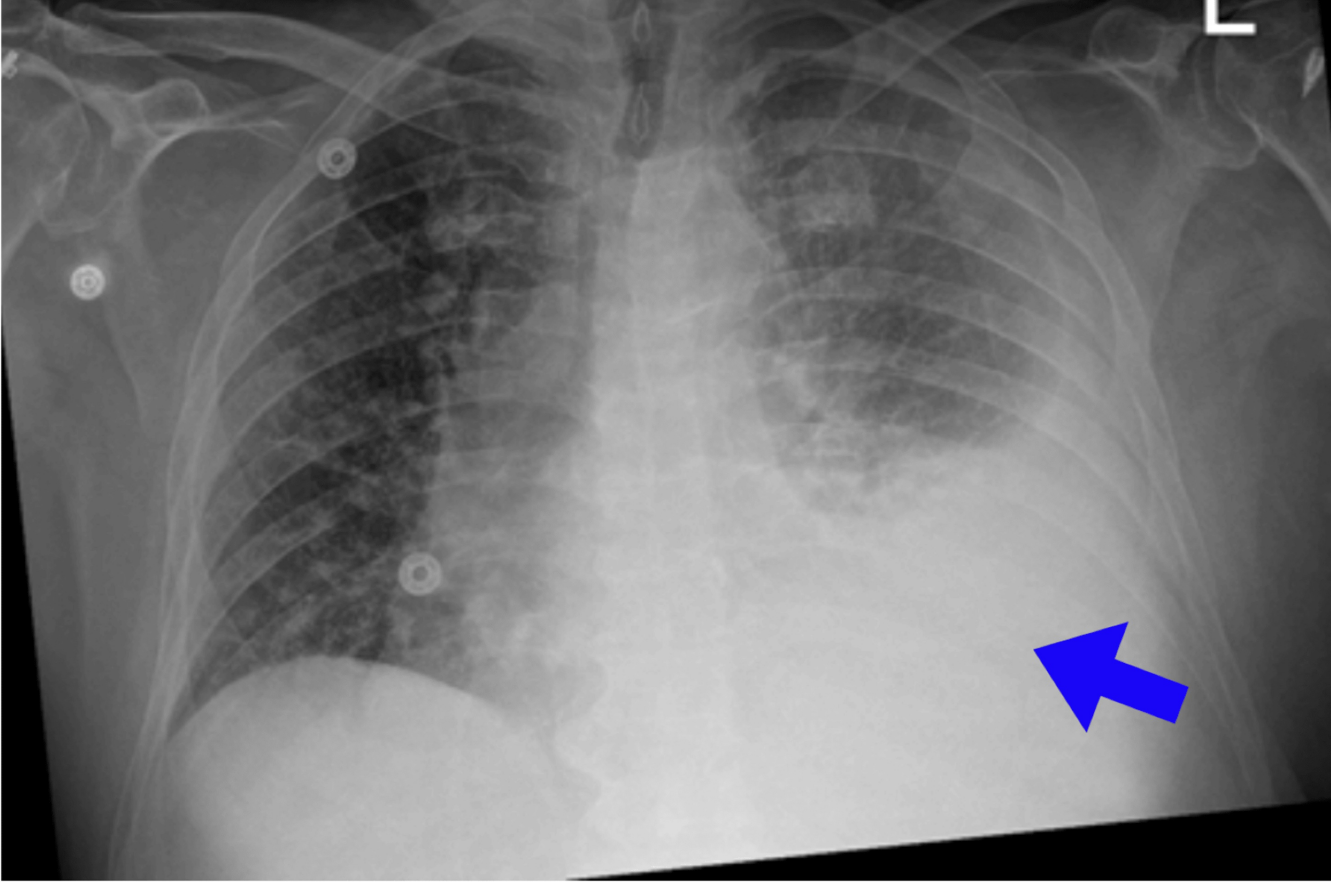
Chest X-ray showing a left-sided pleural effusion

His blood pressure was 108/74 mm Hg, heart rate 84 beats per minute, temperature 98.8 F, respiratory rate 16/minute, and saturation 94% on room air. On physical examination, decreased breath sounds were noted more in the left lung base. Additionally, there was a trace of bilateral lower extremity edema. A chest X-ray revealed a moderate left-sided pleural effusion (Figure 1). There was a normal sinus rhythm with no ST and T wave changes on the ECG. Echocardiography showed normal left ventricular cavity size with normal systolic function and impaired left ventricular relaxation.

The patient underwent 1500 ml of pleural fluid removal from the left side. The pleural fluid analysis revealed exudative pleural effusion (Light's criteria) with lymphocyte predominance in the pleural fluid cell differential. We planned a diagnostic workup to investigate the possibility of malignancy, such as lymphoma, chronic infections like tuberculosis (TB), and inflammatory causes. Despite multiple cytology and cultures returning negative results, the pleural effusion continued to re-accumulate (Table 1). CT chest showed recurrent left-sided pleural effusion, atelectasis in the inferior left perihilar region, and no evidence of significant adenopathy or other soft tissue masses in the chest or visualized abdomen (Figure 1). 

**Table 1:** Pleural fluid analysis from the initial and subsequent thoracentesis LDH: lactate dehydrogenase; pH: potential of hydrogen; WBC: white blood cell; PMN: polymorphonuclear leukocyte

Parameter/date	10/10/23	10/30/23	11/29/23	12/13/23	12/27/23	1/11/24
Protein	3.7	3.5	3.3	3.1	3.2	2.7
LDH	96	106	95	90	105	110
Glucose	124	129	132	120	123	112
Albumin	-	-	2.4	2.3	-	-
pH	7.42	7.46	7.39	7.46	7.46	7.89
WBC	1529	1636	2213	1487	1606	1109
PMN	8	10	4	2	10	7
Lymphocytes	78	74	94	92	76	77
Monocytes	9		1	3	-	6
Mesothelial	1	1	1	2	-	2
Cytology	Negative	Negative	Negative	Negative	Negative	Negative
Culture	Negative	Negative				

**Figure 2:**
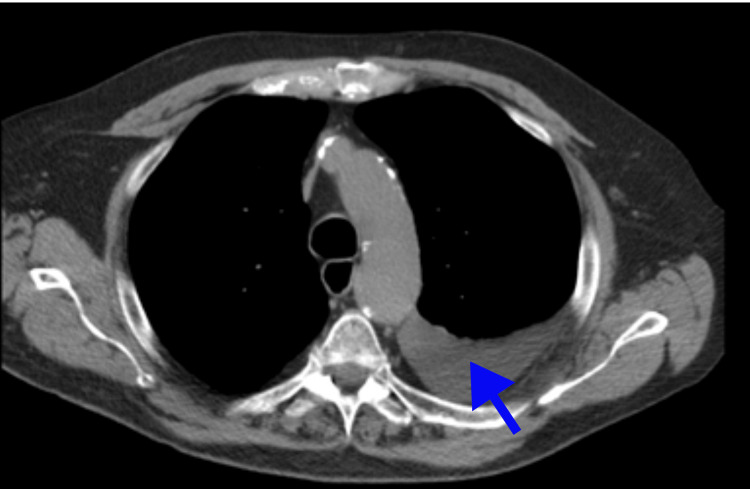
CT chest showing left-sided pleural effusion and no evidence of significant adenopathy or other soft tissue masses

**Figure 3:**
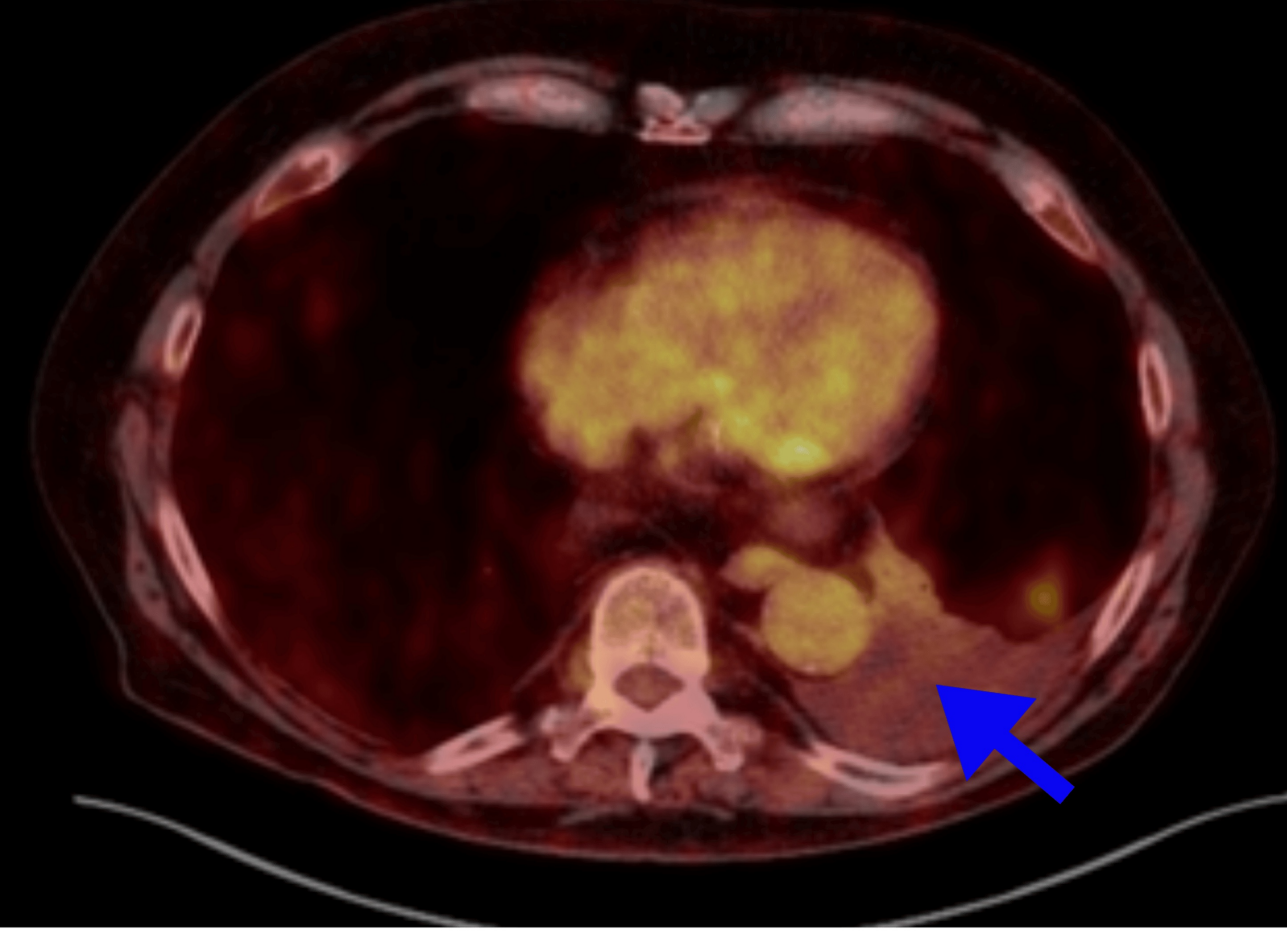
PET scan shows left pleural effusion without metabolically active malignant process

A positron emission tomography (PET) scan revealed small to moderate volume left pleural effusion with adjacent atelectasis, focal pneumonitis in the left lower lung with tree-in-bud opacities, and borderline hypermetabolic activity. There were no definite hypermetabolic findings to confirm a metabolically active malignant process. (Figure 2, 3). There were no ascites or anasarca. We even contemplated performing a pleuroscopy to explore any potential causes that may have been missed given what seemed like an exudative effusion. After closely examining the patient's medical history and re-analyzing the pleural fluid, we found that the serum effusion albumin gradient was found to be greater than 1.2 g/dl. (serum albumin 4.4 g/dl), and it was determined to be pseudoexudate rather than an exudative effusion. We focused on the management of an underlying congestive heart failure with preserved ejection fraction (HFpEF) with optimization of loop diuretics and the addition of a mineralocorticoid antagonist (spironolactone) and an SGLT2 (sodium-glucose cotransporter) inhibitor (empagliflozin). After optimization of HFpEF management, the patient's condition has stabilized, and there has been no need for thoracentesis in the past four months.

## Discussion

Evaluating a patient with pleural effusion can be difficult due to the wide range of possible diagnoses. The 2023 evidence-based guideline from the British Thoracic Society (BTS) offers crucial investigation methods for assessing pleural effusions [3,4]. Pleural fluid is analyzed for general biomarkers such as cell counts and differential, total protein, glucose, lactate dehydrogenase, pleural fluid pH, and cholesterol. Disease-specific pleural fluid biomarkers for infection (pleural fluid culture and gram stain), malignancy (cytology), and chylothorax (lipid analysis) are done on a case-by-case basis [5,6]. Pleural effusions can be transudative or exudative, and the frequency of each type depends on the clinical setting. Table 2 elicits the basic differences between these two [6-9].

**Table 2:** Basic differences between exudative and transudative pleural effusion LDH: lactate dehydrogenase

	Exudative [6-8]	Transudative [6-9]
Mechanism	Enhanced vascular permeability of pleural surfaces or neighboring lung	Alteration of the systemic factors that affect the formation and absorption of pleural fluid (such as hydrostatic and oncotic pressures)
Diagnosis	According to Light's criteria, exudative pleural effusions are identified by one or more of the following: pleural fluid LDH>2/3rd of the upper limit of serum value; pleural fluid to serum LDH ratio >0.60 and LDH >200units in pleural fluid; pleural fluid protein to serum protein ratio >0.50	Exclusion of exudative effusion by Light’s criteria
Causes	Malignant diseases include carcinomas of any origin, particularly those of the lung and breast, mesothelioma, and lymphoma. Infections: parapneumonic effusion; tuberculous pleurisy; fungal, parasitic, or viral infections. Pulmonary embolism autoimmune inflammatory diseases: systemic lupus erythematosus and other connective tissue diseases. Drugs: amiodarone, dasatinib, methotrexate, etc. Intra-abdominal processes: pancreatitis, subphrenic/hepatic abscess. Miscellaneous: traumatic hemothorax, chylothorax, pseudo chylothorax, postcardiac bypass surgery, Dressler syndrome, post-radiation therapy.	Congestive heart failure, liver cirrhosis, nephrotic syndrome, urinothorax, hypothyroidism, hypoalbuminemia

The severity of the symptoms is determined by the effusion's size and the patient's cardiopulmonary reserve. While symptoms related to the underlying cause might be present, pleural effusions typically manifest with nonspecific symptoms such as dyspnea, cough, and chest pain, which can be either dull or pleuritic.

The cause of pleural effusion can be challenging to determine, as it remains unclear in about a quarter of patients despite thorough clinical evaluation and analysis of pleural fluid [3]. Our approach typically involves reviewing the patient’s history and examination, along with a reanalysis of the pleural fluid for recurrent pleural effusion. Although pleural effusion can stem from various etiologies, large studies have shown that cancer, heart failure, and parapneumonic infections are the leading causes. In some cases, the patient may have a preexisting condition that can cause pleural effusion, including systemic lupus erythematosus, hypothyroidism, amyloidosis, pancreatitis, lymphangioleiomyomatosis, rheumatoid arthritis, immunoglobulin G4, drug-induced lupus, and immunotherapy [7].

Multiple case series and meta-analyses have revealed that Light's criteria may effectively rule out exudates. However, specificity is lower than previously reported, with actual transudates misclassified as exudates in 15% to 30% of instances [10,11]. Pleural-to-serum protein gradient, pleural-to-serum albumin gradient, pleural cholesterol, and pleural-to-serum cholesterol ratio are other assays that have been suggested as having a higher specificity [10,11]. Misclassification of transudates using Light's criteria typically happens in patients with heart failure or cirrhosis who are on diuretics [12,13]. Diuretic treatment has been shown in a limited series to increase lactate dehydrogenase and pleural fluid protein, meeting the requirements for an exudate [14]. Similar findings were present in our case. The pleural fluid analysis revealed exudative pleural effusion according to Light's criteria. However, the serum effusion albumin gradient was greater than 1.2 g/dl, and no etiology for exudative effusion was identified. The cause of effusion was determined to be diastolic dysfunction. Such exudative effusions with transudative etiologies are referred to as "pseduoexudates."

Pseudoexudates can be caused by diuretic therapy, traumatic pleural taps, and coronary artery bypass grafting. When determining pseudoexudates, a serum-pleural effusion albumin gradient (SPAG) of >1.2 g/dL and a serum-pleural effusion protein gradient (SPPG) of >3.1 g/dL together showed a sensitivity of 100% in heart failure and a sensitivity of 99% in hepatic hydrothorax [10]. Measuring pleural fluid N-terminal pro-brain natriuretic peptide (NT-proBNP) levels and calculating a serum-to-pleural fluid gradient for albumin or protein can help reclassify the effusion as a transudate. An NT-proBNP level >1500 pg/mL favors the presence of a transudative pleural effusion consistent with heart failure [1,15]. It is possible to have falsely elevated values in patients with septic shock, acute kidney injury, and pneumonia with coexisting heart failure [16]. If an effusion is narrowly characterized as an exudate in a heart failure patient and there are no indications of an underlying exudative cause, treating the condition is reasonable. Further investigations may be necessary if the effusion does not improve.

Cytological testing is essential in suspected cases of malignant pleural effusion. After the first thoracentesis, the sensitivity of pleural fluid cytology in diagnosing malignant effusions is about 60%. However, by obtaining a second thoracentesis sample, the diagnostic yield may increase by 15%. With further attempts, a success rate of up to 90% can be achieved after collecting three samples on separate days [17]. It is important to send at least 75 mL for cytology to enhance diagnostic yield. However, the guideline from the College of Pathologists recommends sending as much fluid as possible [17,18]. In cases of suspected lymphoma, flow cytometry, and immunohistochemical staining are also important in identifying lymphomatous cells. In our case, although lymphocytes were present, the cytology results from the subsequent thoracentesis were negative. Therefore, with the assistance of imaging studies, we were able to rule out active malignancy. In cases of inconclusive imaging and pleural fluid findings, a pleural biopsy is required for the further assessment of malignant pleural changes [5].

When there is suspicion of tubercular effusion, a commonly used diagnostic threshold to confirm a TB pleural effusion is a pleural fluid adenosine deaminase (ADA) level greater than 40 U/L. If the ADA level is less than 40 U/L, it is unlikely to be TB, indicating a high negative predictive value [19,20]. It's important to note that elevated pleural fluid ADA levels can also occur in conditions other than TB, such as bacterial empyema, mesothelioma, lung cancer, lymphoma, parapneumonic effusion, and hematologic malignancies. According to some studies, an ADA level higher than 45 to 60 units/L is considered 100 percent sensitive and up to 97 percent specific for tuberculous pleural effusion [21,22].

Soluble mesothelin-related peptides (SMRPs) are detected in serum or pleural fluid. They are peptide fragments of mesothelin, a glycoprotein present in normal mesothelial cells but found in higher levels in mesothelioma cells. A meta-analysis of 16 diagnostic studies showed that the sensitivity of serum SMRPs varied from 19 to 68 percent [23]. Few studies suggest that SMRP is a valuable marker for mesothelioma in both serum and pleural effusion fluid, supporting further studies of SMRP combined with other markers for screening of asbestos-exposed cohorts [24].

It requires at least 75 mL of fluid to obliterate the posterior costophrenic sulcus and a minimum of 175 mL to obscure the lateral costophrenic sulcus on an upright chest radiograph. CT imaging can detect very small pleural effusions, even those less than 10 mL, and possibly as minimal as 2 mL of fluid in the pleural cavity [25]. The observation of thickened and enhanced visceral and parietal pleura following intravenous contrast administration referred to as the 'split pleura sign,' typically indicates inflammation, pointing to an exudative effusion. Moreover, CT scans can reveal lung parenchymal issues that large pleural effusions might obscure on chest radiographs and can assist in guiding thoracentesis and tube thoracostomy for loculated empyema [25]. Ultrasonography is useful for detecting both free and loculated pleural effusions and can distinguish loculated effusions from solid masses. Ultrasound guidance also aids in the thoracentesis of loculated pleural effusions. However, for more complex interventional procedures, like empyema drainage or pleural mass biopsies, CT remains the preferred method [26]. Fluorine-18-fluorodeoxyglucose positron emission tomography (FDG-PET) scanning has demonstrated limited accuracy in distinguishing between malignant and benign pleural effusions. Although it can differentiate exudative from transudative pleural effusions, it is not commonly recommended for distinguishing benign from malignant pleural effusions [27].

Treatment

Treat Primary Disorder

The initial management of symptomatic nonmalignant pleural effusion (NMPE) focuses on addressing the underlying cause and draining the pleural effusion. Treatment approaches differ based on the specific etiology. They may include diuretics for heart failure, antibiotics for pneumonia, diuresis, and transjugular intrahepatic portosystemic shunt (TIPS) for hepatic hydrothorax, non-steroidal anti-inflammatory drugs (NSAIDs) for lupus pleuritis, or ultrafiltration for fluid overload in renal failure patients [28,29]. In this case, despite initial concerns for malignant pleural effusion, thorough evaluation, including fluid analysis, imaging, and echocardiography, led to the diagnosis of a pseudoexudate likely influenced by diuretic therapy for heart failure. The patient's pleural disease stabilized with the administration of diuretics, reducing the need for repeated thoracentesis.

Pleural Fluid Drainage for Symptomatic Patients

Drainage procedures are recommended for patients with symptomatic NMPE. The BTS recommends ultrasound-guided thoracocentesis to minimize the risk [3]. Generally, the safe volume of fluid that can be removed remains uncertain, but it is commonly suggested that 1 to 1.5 liters should be the maximum amount extracted in a single session to reduce the risk of complications like re-expansion pulmonary edema (RPE). However, some evidence indicates that RPE may not be related to the volume of fluid removed [30]. If the symptomatic NMPE persists or recurs, the preferred first-line treatment is repeat therapeutic thoracentesis under ultrasound guidance [3].

Management of First Recurrent or Persistent Pleural Effusion

The majority of NMPE patients respond well to primary disorder treatment and drainage. Still, a considerable proportion persist or reoccur. In these patients, before proceeding to definitive therapy to prevent recurrences like pleurodesis or indwelling pleural catheter (IPC) placement, it is recommended to redrain symptomatic pleural effusions, confirm the underlying cause of the NMPE, and rule out nonexpendable lung disease, pleural infection, hepatic hydrothorax, and malignancy [3].

Refractory Pleural Effusion

Patients who are experiencing symptomatic NMPEs that continue to recur despite undergoing multiple thoracentesis procedures (for example, more than two to three) and receiving the best available medical treatment should be evaluated for placement of an indwelling pleural catheter (IPC) and/or pleurodesis. It is important to note that pleuroperitoneal or pleurovenous shunts, as well as surgical pleurectomy, are considered last-resort options and are seldom utilized or necessary.

For patients with suspected complicated parapneumonic effusion (CPPE), an intercostal drain (ICD) should be implanted if the volume of accessible pleural fluid on ultrasound indicates that it is safe to do so [4,5]. For malignant pleural effusion, the guidelines advise against delaying definitive pleural intervention until after systemic anti-cancer therapy (SACT). When treating malignant pleural effusions (MPE), talc pleurodesis (or an alternative technique) is advised over recurrent aspiration, particularly when the prognosis is favorable. When treating malignant pleural effusion (MPE), patients who do not have a known non-expandable lung should be offered the choice of pleurodesis or an indwelling pleural catheter (IPC) [3,4].

## Conclusions

Evaluating the cause of pleural effusion is sometimes challenging, and conventional diagnostic tests may be misleading. Light’s criteria provide important diagnostic clues, but sometimes it could misclassify the pleural effusion. Serum-effusion albumin gradient is a reliable criterion for differentiating exudative from transudative effusion when there is clinical suspicion. In cases of false exudates from a cardiac origin, an albumin gradient >1.2 g/dL is the most suitable parameter. This case illustrates the complex presentation and management challenges of recurrent unilateral pleural effusions and underscores the importance of comprehensive and stepwise systematic diagnostic approaches and personalized management strategies.
